# Impact of Low Dose Prophylaxis (LDP) on spontaneous bleeds in Hemophilia-A: A Bicentre study from Pakistan

**DOI:** 10.12669/pjms.42.4.12279

**Published:** 2026-04

**Authors:** Maryam Khan, Tahira Zafar, Lubna Zafar, Ayesha Imran, Shahla Tariq Sohail

**Affiliations:** 1 Maryam Khan, Consultant Clinical Hematologist, Armed Forces Bone Marrow Transplant Center, CMH Medical Complex, Rawalpindi, Pakistan; 2 Tahira Zafar, Consultant Hematologist, Director Hemophilia Treatment Centre, Rawalpindi, Pakistan; 3 Lubna Zafar, Professor of Hematology, Director Hemophilia Treatment Centre, Rawalpindi, Pakistan; 4 Ayesha Imran, Consultant Hematologist, Hemophilia Patient Welfare Society, Lahore, Pakistan; 5 Shahla Tariq Sohail Patron, Pakistan Hemophilia Patients Welfare Society, Lahore, Pakistan

**Keywords:** Bleeding, Hemophilia, Low dose prophylaxis

## Abstract

**Background & Objectives:**

Hemophilia-A is a bleeding disorder characterized by musculoskeletal bleeding. Standard dose factor replacement (SDP) is the mainstay of treatment for severe or moderately severe HA patients however it is fraught with increased cost and burden on health care system. Low dose prophylaxis (LDP) has gained popularity in low- and middle-income settings (LMIC) as being more feasible and cost effective. The aim of this study was to establish efficacy of low dose prophylaxis of clotting factor concentrates (CFCs) as primary or secondary prophylaxis strategy on the bleeding outcomes of pediatric severe HA patients.

**Methodology:**

This bicentre prospective collaborative study from the Hemophilia Patient Welfare Society (HPWS) and the World Federation of Hemophilia (WFH) commenced at Rawalpindi & Lahore Hemophilia Treatment Centers (HTCs) in Pakistan from January 2017 to January 2023.This study assessed the annualized bleeding rates (ABR) before and after LDP in pediatric HA patients as primary or secondary prophylaxis.

**Results:**

This bicenter study from Lahore and Rawalpindi reports statistically significant reduction in ABR after LDP (p=<0.0001) in 46 pediatric male patients. The study reports good compliance, reduced ABR and no development of new inhibitors in these patients all followed for one year post LDP start.

**Conclusion:**

This study shows the efficacy of LDP in pediatric HA patients in reducing ABR. This also gives a safety signal in terms of no major breakthrough bleeds and no new inhibitor formation.

## INTRODUCTION

Hemophilia-A (HA) is an X-linked congenital bleeding disorder characterized by deficiency of factor VIII (FVIII).^1^ It is more common in males and effects one in every 5000-10,000 new born male infants.^2^ Severe Hemophilia-A is characterized by recurrent spontaneous bleeds particularly musculoskeletal and joint bleeds.^3^ Plasma derived and recombinant FVIII replacement has been the corner stone of management for years.^1^ The cost and access to the clotting factor concentrates (CFCs) remains a challenge in under resourced settings.^4^

Frequent infusions of high dose CFCs as standard dose prophylaxis(SDP) at 25-40IU/kg alternate day have been employed and resulted in reduced annualized bleeding rates(ABR), hemophilia joint health score (HJHS) and quality of life (QoL).^5^ However this approach has been associated with significant burden on economy.^4^ Hemophilia treatment modalities include on-demand or continuous treatment including primary, secondary and tertiary prophylaxis.^6^ The World Federation of Hemophilia(WFH) reported almost two million people form 115 countries in 2019 out of which almost 2000 patients were from Pakistan, however the exact incidence remains unknown. In low and middle income countries including Pakistan, the traditional high dose approach is not feasible and low dose and very low dose prophylaxis treatment (PT) has shown to be effective and associated with better compliance.^7^

We aimed to assess the effect of LDP on the ABR on spontaneous bleeds of HA pediatric patients one year before and one year after the beginning of LDP intervention. We hypothesized that the mean difference in the ABR on spontaneous bleeds – herein referred to as ABRs – between before and after LDP would be different than zero in favor of LDP.

## METHODOLOGY

A quasi-experimental time-series design was used to assess ABR during a six month period before and a one year period after the introduction of LDP. This real-world study investigated the efficacy of primary and secondary prophylaxis with low dose Factor VIII (FVIII) concentrate in pediatric patients up to age 10 years with Hemophilia-A without inhibitors in Pakistan. This collaborative study between the Hemophilia Patient Welfare Society (HPWS) and the World Federation of Hemophilia (WFH) commenced on January 2017 at Rawalpindi & Lahore Hemophilia Treatment Centers (HTCs) in Pakistan and lasted until January 2023. This study was conducted at the Hemophilia treatment centers Rawalpindi and Lahore, Pakistan from January 2017 to January 2023.Patients were observed for a period of up to two years.

FVIII concentrates were provided by WFH on donation basis for low middle income countries. Quality assurance, maintenance of cold chain, appropriate storage and administration of products were ensured by HPWS and HTCs in accordance with principles defined by World Health Organization (WHO) and WFH. The patient data was entered and analyzed in the WFH World Bleeding Disorders Registry.

### Ethical Approval:

The study protocol was approved by Institutional Review Board (IRB) of HPWS (letter number DH/TZ/IRB-HTC-03 dated February 27, 2017). Informed written consent was obtained from parents/guardians of all participants prior to inclusion

### Inclusion Criteria:

Patients (less than 10 years of age), with severe Hemophilia-A without inhibitors, and who lived proximal to the HTC were included in this study. Patients had to have healthy joints as defined by Hemophilia Joint Health Score (HJHS) of at least 80-90% assessed by an orthopedic surgeon/physiotherapist prior to entry.

### Exclusion Criteria:

Patients with a transfusion transmitted viral infection (HbsAg positive, anti HCV & PCR & anti-HIV positive), or history of major bleeding as defined by ISTH^8^ were not included.

### Dosage of LDP:

All patients had a starting dose of FVIII concentrates of 250 IU once a week for extended half-life (EHL) products and 250 IU twice weekly for short half-life (SHL) products as per WFH protocol for the study. To be included, patients had to remain on CFCs for at least six months.

Bleeding that occurred while on prophylaxis (breakthrough bleeds) were treated as per WFH guidelines^5^ until full resolution before resuming prophylaxis. If further joint bleeds occurred, dose was increased to 250 IU three times a week for SHL product and 250 IU twice a week for EHL product. If no further bleeds occurred, this dose was maintained.

### Data collection:

Patients were reviewed at clinic fortnightly. All data were entered into the World Bleeding Disorders Registry (WBDR). Patient demographics were noted at baseline and number of spontaneous bleeding events were recorded at each clinic visit. All data were downloaded from the WBDR for analysis. To ensure compliance, patients were regularly followed up at our outpatient’s clinic and regular telephonic contacts were maintained with families. Inhibitors to clotting factors were screened for six monthly. Adverse reactions and hemophilia joint health score(HJHS) were measured every three months.

### Statistical Analysis:

Data processing and statistical analyses were carried out using R 4.2.1 (R Core Team, 2022), and stats, tidyverse (Wickham et al., 2019), eeptools (Knowles, 2023), arsenal (Heinzen et al., 2021) and basictabler (Bailiss, 2021) packages.

The total number of spontaneous bleeds in the six months prior to start of prophylaxis were multiplied by two for annualization. Likewise, the total number of spontaneous bleeds in the one year after the first LDP infusion was calculated from data from clinic visits entered in the WBDR. These two records were the ABRs before and after LDP, respectively. All patients spent a full year in ongoing LDP after the first infusion, except for seven. For the patients with treatment interruption, time on LDP was calculated by subtracting the interruption interval in days from 365.

Age at start of LDP range and central tendencies (means ± standard deviations (SD) or medians with interquartile ranges – referred to as ‘Q1 – Q3’ or ‘IQR’) were summarized. Additionally, ABRs central tendencies of the one year before and the one year after LDP were summarized. To address our hypothesis that the mean difference between ABRs before versus after LDP is different than zero, a paired t-test with a significance value of α=0.05 was conducted, after fulfilling the normality assumption of the distribution of the differences in ABRs with a Shapiro-Wilk test (W = 0.98, p=0.67).

## RESULTS

This study included 46 male patients who fulfilled the study criteria. The mean age at start of LDP was 4 ± 2 SD years (range 1-9 years) (Table-I). The mean LDP dose was 20.37 IU/kg/ wk (range 8.06 - 35.7 IU/kg/wk). Thirty-nine (85%) patients completed one year of continuous LDP as of the start of the intervention (Table-I). Due to a lack of available LDP at the time, seven patients had a treatment interruption that lasted 47 days, being on LDP for 10.5 months. No patient developed inhibitors.

**Table-I:** Summary of the Demographics and LDP.

	N=46*
***Type of hemophilia, n(%)***	
A	46 (100%)
Gender, n(%)	
Male	46 (100%)
***Age as of patient’s first LDP infusion, years***	
Median (Q1, Q3)	4 (3, 6)
Mean (SD)	4^2^
Range	1 – 9
***Patient completed the first year of continuous LDP, n(%)***	
No	7 (15%)
Yes	39 (85%)

* Number of unique paediatric patients on LDP treatment included in the study.

**Fig.1:**
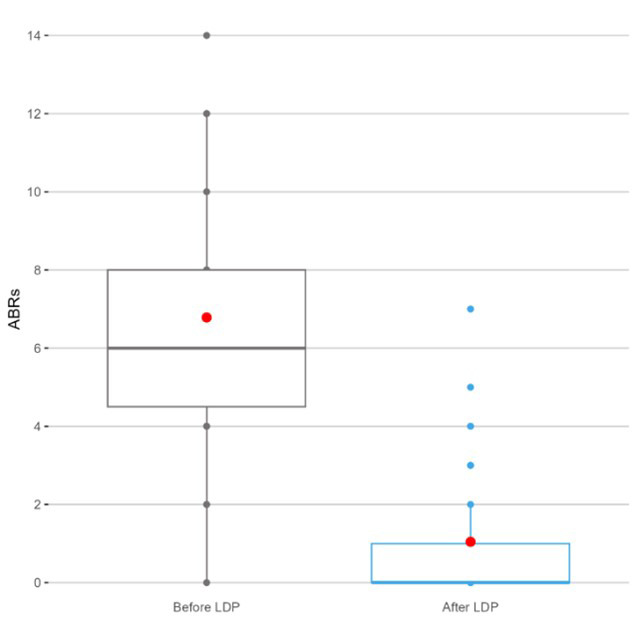
Annualized bleeding rates on spontaneous bleeds (ABRs), median (IQR), before and after LDP. The mean ABRs are represented by a red circle.

**Fig.2:**
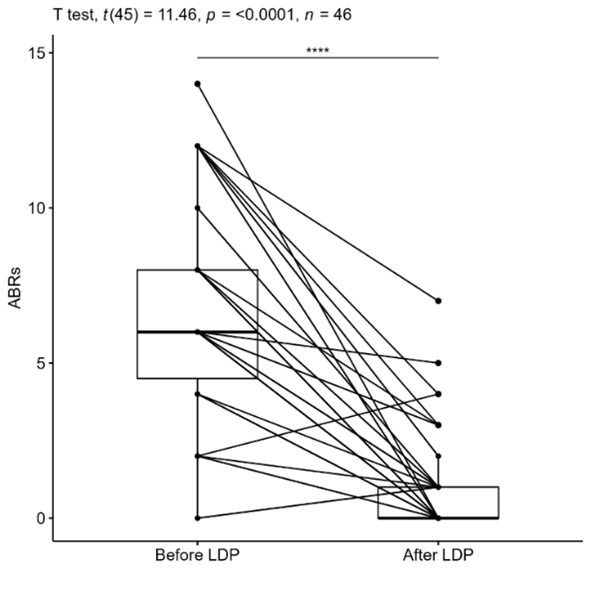
Paired t-tested representation of the ABRs before vs after LDP.

The median ABRs reduced from six before LDP to 0 after LDP (Table-II, Fig.1). The mean difference (MD) ABRs was significantly different than 0 (t(45)=11.463, p=<0.0001), with patients’ ABRs before LDP being on average six bleeds higher (MD=6, 95% confidence interval 5-7) than after the treatment (Fig.2).

**Table-II:** Summary of ABRs in the one year before and the one year after the LDP intervention, for the total of 46 severe HA pediatric patients.

	Before LDP (N=46*)	After LDP (N=46*)
***Annualized bleeding rate on spontaneous bleeds (ABRs)***		
Median (Q1, Q3)	6 (4, 8)	0 (0, 1)
Mean (SD)	7 ^(3)^	1 ^(2)^

* Number of unique paediatric patients on LDP treatment included in the study.

## DISCUSSION

This study described the real-world data of pediatric patients treated on LDP program supported by WFH in two cities of Pakistan, with a primary focus on ABR and spontaneous bleeds. The results show the efficacy and feasibility of LDP and found significant reduction in ABR. None of the patients developed inhibitors which is encouraging. Also, the compliance to treatment was 100%. No patients had significant issues with vascular access and peripheral venous cannulas were used for all.

There was treatment interruption of almost 47 days due to non-availability of CFCs temporarily. This however fortunately did not lead to any negative impact on ABR. Patients were called for resumption of LDP as soon as CFCs became available.

Our study concurs with the findings of other studies supporting LDP especially in LMIC settings. This approach is highly feasible especially effective if started earlier. This approach leads to significant cost reduction and also avoids major complications associated with HA including but not limited to bleeding events and inhibitor formation.^9^,^10^ Vascular access difficulty leading to use of central access device or other implantable catheters which has traditionally been a major problem in pediatric PHA was not encountered in our population.^11^ This could be due to placement of dedicated nurses and phlebotomist for IV cannulation and avoiding frequent change in staff. This not only led to experience personnel performing cannulation but also rapport development between child and the person administering LDP. This probably led to successful cannulation allowing delivery of LDP effectively. Our cohort consisted of younger patients with baseline healthy joints and studies have suggested that earlier age of onset and baseline healthy joint are important predictors of improved response.^12^ Another major strength of the study is the compliance of patients and carers. This was made possible by patient and carer education, frequent in person and telephonic contact of HTC staff with patients and their families. There was also a lot of emphasis on patient education and lifestyle modification to avoid trauma which could have led to lower incidence of hemostatic challenge. Earlier reporting to HTC in case of break through bleeding was emphasized and although break through bleeds did occur, none of them were clinically significant or required factor replacement. Inhibitor development was not seen in any of the patients and this re enforces the efficacy of LDP in prevention of inhibitor formation which are most frequently associated with episodic treatment(ET) and tissue trauma.^13^

Extensive work has been published regarding efficacy of LDP. Low dose prophylaxis with FVIII concentrates was first studied in Tunisia using FVIII concentrates at 30 iu/kg/week as secondary or tertiary prophylaxis and reported improved HJHS, FISH and quality of life (QoL).^14^ A study from China showed secondary low dose prophylaxis at dose of 10u/kg twice weekly significantly improved joint functions.^15^ Verma et al also reported that LDP at dose of 10 units /kg body weight twice weekly resulted in significant reduction in hemarthrosis, FVIII consumption and school absenteeism.^16^ Data from Iran also suggests low dose escalating prophylaxis according to patient phenotype is suitable for low income resource countries.^17^ Other studies also reported similar findings. In almost all of these studies, comparison was made between LDP and episodic treatment (ET).^18^ There is no prospective trial comparing standard dose prophylaxis(SDP) and LDP and little data on comparison of intermediate dose prophylaxis(IDP) and SDP.^19^ Although these studies reported improved ABR with LDP compared to ET, data on long term outcomes are sparse. Retrospective data from China suggest that primary prophylaxis group was most benefitted by LDP strategy and age at onset of prophylaxis seems to be significantly associated with better joint health.^20^ Joint outcomes seem to be different across different joints in a retrospective study where best outcomes were seen in knee and elbow and not in ankle joint.^21^ Similarly, dose escalation LDPs have also been employed based on escalation either on basis of clinically significant bleeds^22^ and have resulted in improvement in bleeding outcomes. On the contrary, pharmacokinetic monitoring of trough levels to ensure adequate levels to individualized LDP prophylaxis has also been done.^23^,^24^ It is still uncertain which is the most suitable and cost effective way to reduce ABR, AJBR, FVIII utilization, improved QoL in LMIC settings. Our study adds to the already existing body of evidence suggesting safety, feasibility and improved outcomes of primary LDP in LMIC settings.

### Strengths of the study:

It provides real world prospective data from Pakistan reflecting real practice in under sourced settings. Another strength is that this study focuses on young Hemophilia-A patients with baseline healthy joints establishing a clear assessment on impact of LDP on joint bleeds.

### Limitations:

It includes lack of control arm. Another limitation is bleeding outcomes were assessed clinically and imaging modalities such as ultrasound or MRI were not performed to detect subclinical joint bleeds. Third, although the follow-up period allowed evaluation of short-term outcomes, longer follow-up is needed to assess sustained joint protection and functional outcomes. Finally, the sample size was relatively small and drawn from two centers, which may limit generalizability.

## CONCLUSION

This study emphasizes how close patient contact and follow-up can result in excellent outcomes even in resource limited settings. Since no patient developed inhibitors or major breakthrough bleeds, this also highlights the safety of this approach and applicability in LMIC settings where testing for inhibitors on regular basis is associated with significant cost. Additionally, the use of peripheral cannulas for all patients helped avoid vascular complications. Finally, the use of standardized registry platform (WBDR) ensured uniformity of data entry, longitudinal follow up and external validation.

### Further recommendations:

Areas requiring further study include direct comparison with on demand, intermediate and standard dose prophylaxis regimens. Other areas for study include incorporation of joint imaging (ultrasound and MRI) which may show subclinical bleeding in LDP arm which may impact future joint health. Long term safety data is also needed to establish whether LDP provides durable joint protection and functional outcomes compared to standard dose prophylaxis. Further studies should report health-economic outcomes and cost effectiveness of this technique in comparison to standard prophylaxis arm. More studies from Pakistan should also focus on cost effective ways for management of Hemophilia.
